# Comparative Oncology: Cross-Sectional Single-Cell Transcriptomic Profiling of the Tumor Microenvironment Across Seven Human Cancers

**DOI:** 10.3390/cancers17213527

**Published:** 2025-10-31

**Authors:** Riku Okamoto, Kota Okuno, Akiko Watanabe, Kanako Naito, Hiroyuki Minoura, Shumpei Shibaki, Kyonosuke Ikemura, Keiko Oki, Yu Kuroda, Shiori Fujino, Yusuke Nie, Nobuyuki Nishizawa, Eiichiro Watanabe, Mariko Kikuchi, Koshi Kumagai, Takahiro Yamanashi, Hiroshi Katoh, Hajime Takayasu, Takeo Sato, Takafumi Sangai, Yusuke Kumamoto, Takeshi Naitoh, Naoki Hiki, Keishi Yamashita

**Affiliations:** 1 Division of Advanced Surgical Oncology, Research and Development Center for New Medical Frontiers, Kitasato University School of Medicine, Kitasato 1-15-1, Minami-ku, Sagamihara 252-0374, Kanagawa, Japan; okamoto.riku@kitasato-u.ac.jp (R.O.);; 2 Department of General-Pediatric-Hepatobiliary Pancreatic Surgery, Kitasato University School of Medicine, Kitasato 1-15-1, Minami-ku, Sagamihara 252-0374, Kanagawa, Japan; 3 Department of Breast and Thyroid Surgery, Kitasato University School of Medicine, Kitasato 1-15-1, Minami-ku, Sagamihara 252-0374, Kanagawa, Japan; 4 Department of Upper Gastrointestinal Surgery, Kitasato University School of Medicine, Kitasato 1-15-1, Minami-ku, Sagamihara 252-0374, Kanagawa, Japan; 5 Department of Lower Gastrointestinal Surgery, Kitasato University School of Medicine, Kitasato 1-15-1, Minami-ku, Sagamihara 252-0374, Kanagawa, Japan

**Keywords:** comparative oncology, single-cell RNA sequencing, tumor microenvironment, cancer heterogeneity, stromal cell, epithelial cell, cancer-associated fibroblasts

## Abstract

**Background/Objectives**: To elucidate the differential transcriptional and intercellular signaling features of tumor components across various cancers, we conducted a comparative analysis using single-cell RNA sequencing (scRNA-seq). This technology enables detailed characterization of tumor ecosystems and may explain variations in tumor behavior among distinct cancer types. **Methods**: We analyzed publicly available scRNA-seq datasets (GEO) from seven cancer types—pancreatic ductal adenocarcinoma (PDAC), hepatocellular carcinoma (HCC), esophageal squamous cell carcinoma (ESCC), breast cancer (BC), thyroid cancer (TC), gastric cancer (GC), and colorectal cancer (CRC)—to define their unique molecular characteristics and intercellular interactions. **Results**: PDAC displayed a distinct tumor microenvironment (TME) dominated by myeloid cells (~42%), including abundant *CXCR1*/*CXCR2*-expressing tumor-associated neutrophils (TANs) that preferentially interacted with immune rather than cancer cells. The competitive receptor *ACKR1* was minimally expressed on endothelial cells, consistent with PDAC hypo-vascularity. In HCC, tumor cells lacked *EPCAM* and expressed complement and stem cell markers; cancer-associated fibroblasts (CAFs) were scarce, and stellate cells expressed the pericyte marker *RGS5*. CAFs were abundant in ESCC and BC, with *IGF1*/*2* expression, while in GC, these markers were uniquely found in plasma cells. Since BC and GC subtypes exhibit distinct TME patterns, it is necessary to perform subtype-specific analyses for these cancers. TC showed high expression of tumor-suppressor genes, including *HOPX*, in tumor cells. Differential interactions and the presence of “dominant signaling cell populations “ with dominant outgoing signals may underlie the heterogeneity in tumor aggressiveness across these cancers. **Conclusions**: Comparative scRNA-seq analysis of multiple cancers reveals distinct tumor phenotypes and cell–cell communication patterns, offering insights into the molecular architecture of human solid tumors.

## Simple Summary

Cancer is not composed of a single cell type but rather a complex community of cancer cells, immune cells, and supporting stromal cells that communicate with each other. These cellular conversations shape how each cancer grows, spreads, and responds to treatment. In this study, we compared single-cell sequencing data from seven different human cancers to explore how these cell interactions differ among tumor types. We found that pancreatic cancer contains many neutrophils, a type of immune cell that interacts mainly with other immune cells, while liver cancer lacks the usual fibroblast support cells. In contrast, esophageal and breast cancers were rich in fibroblasts that send growth signals to tumor cells, and thyroid cancer retained genes that may slow tumor progression. These differences help explain why some cancers behave more aggressively than others. Our findings provide a clearer picture of how the tumor environment varies among cancers and may guide the development of new strategies to treat solid tumors by targeting their surrounding cells.

## 1. Introduction

Cancer is a leading cause of death globally, and its biological complexity continues to pose major challenges for the development of effective therapies. While traditional bulk-tumor analyses have provided important insights, they often overlook the cellular heterogeneity and dynamic intercellular interactions within the tumor microenvironment (TME), which are key drivers of cancer progression, metastasis, and immune evasion [1].

Recent advances in single-cell RNA sequencing (scRNA-seq) technology have enabled high-resolution dissection of tumor ecosystems, allowing for the identification of novel cell populations and signaling pathways that underlie tumor heterogeneity [2,3]. This approach has highlighted the importance of stromal and immune components in modulating the TME, yet most studies have focused on single cancer types, limiting our understanding of shared versus cancer-specific features.

Certain tumor types, such as pancreatic ductal adenocarcinoma (PDAC) and hepatocellular carcinoma (HCC), display especially distinctive microenvironments: PDAC is enriched in tumor-associated neutrophils (TANs) with immunosuppressive phenotypes, whereas HCC lacks cancer-associated fibroblasts (CAFs) and instead features pericyte-like stellate cells [4,5]. In contrast, esophageal squamous cell carcinoma (ESCC) and breast cancer (BC) exhibit abundant CAFs that shape local signaling networks and immune landscapes [6]. These variations suggest that comparative analysis across cancer types may reveal conserved or divergent principles of TME organization, potentially providing insights into biomarker development or therapeutic approaches targeting the TME.

To address this knowledge gap, we conducted a comparative scRNA-seq analysis of seven human cancers, focusing on intercellular signaling within the TME. Our findings reveal both conserved and cancer-specific stromal and immune architectures, offering novel insights into tumor biology and potential avenues for targeted therapeutic strategies in surgical oncology. We selected these seven cancer types to capture a wide range of biological and clinical diversity. In broad clinical terms, thyroid cancer (TC) and BC are generally associated with more favorable prognoses, whereas PDAC, ESCC, and gastric cancer (GC) are typically characterized by more aggressive behavior. Colorectal cancer (CRC) represents an intermediate malignancy in terms of progression and treatment outcome. Notably, HCC often spreads intrahepatically and rarely metastasizes to lymph nodes, making it distinct from the others. This balanced selection was intended to reflect diverse tumor microenvironmental contexts and facilitate meaningful cross-cancer comparisons.

## 2. Materials and Methods

A schematic overview of the analytical workflow is shown in Supplementary Figure S1, which outlines (a) the single-cell transcriptomic analysis across seven cancer types and (b) the survival analysis using the GSE15459 GC dataset.

### 2.1. Single-Cell RNA-seq Data Processing

Publicly available scRNA-seq datasets were obtained from the Gene Expression Omnibus (GEO) under the following accession numbers: CRC (GSE200997 [7]), BC (GSE176078 [8]), GC (GSE183904 [9]), TC (GSE184362 [10]), PDAC (GSE155698 [11]), HCC (GSE151530 [12]), and ESCC (GSE160269 [13]) (Table S1). Raw data were processed using standard workflows implemented in Seurat (version 4.3.0, R version 4.4.2; R Foundation for Statistical Computing, Vienna, Austria). For CRC, a pre-integrated matrix was used, whereas other datasets were manually integrated following quality control and normalization. Cells were filtered based on gene count, unique molecular identifier (UMI) thresholds, and mitochondrial gene content using cancer-type-specific quality-control criteria. In general, cells with 200–2500 detected genes and <10% mitochondrial transcripts were retained, except for PDAC (mitochondrial threshold 6.5%) and ESCC (minimum UMI count 500).

Doublets were identified and removed using DoubletFinder [14] (version 2.0.4). This step was applied to five cancer types (PDAC, HCC, BC, GC, and TC), whereas CRC and ESCC were excluded because their data were pre-integrated or population-sorted. The expected doublet rate was set at 7.5% for most datasets and 10% for BC to improve cluster separation. The pK parameter was optimized for each dataset by parameter sweep analysis, while pN was fixed at 0.25. Batch correction was then performed using Harmony (version 1.2.3) applied after doublet removal to minimize technical variation across samples while preserving biologically relevant structure.

Dimensionality reduction was performed using principal component analysis (PCA) based on the top 10 principal components, followed by graph-based clustering (resolution = 0.5) and Uniform manifold approximation and projection (UMAP) visualization. A uniform resolution of 0.5 was applied across all cancer types to maintain comparable cluster granularity, and this setting produced cell-type segregation consistent with canonical marker gene expression.

### 2.2. Cell Type Annotation

Cell type annotation was performed by reference-based manual curation using canonical marker gene expression patterns. Major tumor and stromal populations were identified as follows: cancer cells (*EPCAM*, *KRT18*); T cells and subpopulations (*CD3E*, *CD8A*, *FOXP3*); endothelial cells (*PECAM1*, *RAMP2*); pericytes (*RGS5*); and CAFs (*DCN*, *C1S*, *CXCL12*, *COL12A1*). B-type tumor-infiltrating lymphocytes (B TILs) (*MS4A1*), mast cells (*KIT*), myeloid cells (*CD14*), and plasma cells (*MZB1*) were also defined.

For clusters lacking clear marker expression, differentially expressed genes were calculated using Seurat’s FindAllMarkers() function, and the resulting marker profiles were compared with known cell-type signatures reported in previous tumor single-cell studies to confirm annotation consistency. This reference-guided manual approach ensured biologically coherent identification of major cell types across all cancer types.

### 2.3. Cell–Cell Communication Analysis

Cell–cell communication analysis was performed for each cancer type using CellChat [15] (version 1.6.1). Normalized expression matrices and unsupervised cluster annotations were used to construct CellChat objects. We focused on the “Secreted Signaling” category, which primarily reflects paracrine and autocrine communication within the TME and was most relevant to our comparative objectives. Other categories (e.g., ECM–receptor and cell–cell contact) were not included to avoid redundancy and to maintain a uniform analytical framework across datasets. Overexpressed interactions and communication probabilities were computed using standard CellChat functions (e.g., identifyOverExpressedInteractions, computeCommunProb) and visualized using circular network diagrams (netVisual_circle). Communication probabilities were compared qualitatively across cancers based on relative pathway activity rather than absolute numerical values.

### 2.4. Survival Analysis Using Gene Expression Data

For survival analysis, we used the publicly available dataset GSE15459, which represents a well-annotated GC cohort with long-term clinical follow-up data [16] (Table S2). We selected this dataset because it provides high-quality microarray profiles from 200 primary gastric adenocarcinomas, allowing for the reproducible validation of gene expression–survival associations. Among the seven analyzed cancer types, GC was chosen as a representative cohort for prognostic evaluation because *CXCR2*^+^ myeloid cells were absent in GC, enabling assessment of the prognostic significance of *TREM2* without confounding by overlapping myeloid subtypes.

Raw CEL files were normalized using the robust multi-array average (RMA) method implemented in the affy package in R. To maintain consistency with our prior analyses and reduce inter-platform variability, expression levels were further adjusted relative to *GAPDH* (probe 213453_x_at), which showed the most stable and correlated signal among candidate *GAPDH* probes. This normalization provided a relative expression index that facilitated cross-gene comparison under the same analytic framework.

Expression values were then imported into JMP Pro (version 18.0.2; SAS Institute Inc., Cary, NC, USA) for downstream analysis. Receiver operating characteristic (ROC) analysis was applied to determine optimal dichotomization cutoffs for overall survival, and Kaplan–Meier curves were generated accordingly. These analyses were exploratory and not intended for independent prognostic modeling; therefore, no multiple testing correction was applied.

### 2.5. Statistical Analysis

Statistical analyses were performed using R and associated packages. For single-cell data analyses, statistical significance at the gene or pathway level was evaluated using the default methods implemented in Seurat and CellChat, both of which apply Benjamini–Hochberg false discovery rate (FDR) correction for multiple testing. Comparisons across cancer types were conducted in an exploratory and descriptive manner without formal statistical modeling, as each dataset was independently processed. For survival analysis, statistical testing was performed as described above.

### 2.6. Declaration of Generative AI and AI-Assisted Technologies in the Writing Process

An AI-based tool (ChatGPT by OpenAI, GPT-4, accessed March 2025) was used under author supervision in a limited manner to improve English expression, clarify structure, and adjust word count. No payment was made for this assistance. The authors reviewed and edited the content independently and took full responsibility for the final manuscript.

## 3. Results

### 3.1. UMAP-Based Comparison of Seven Human Cancers

**Figure 1:**
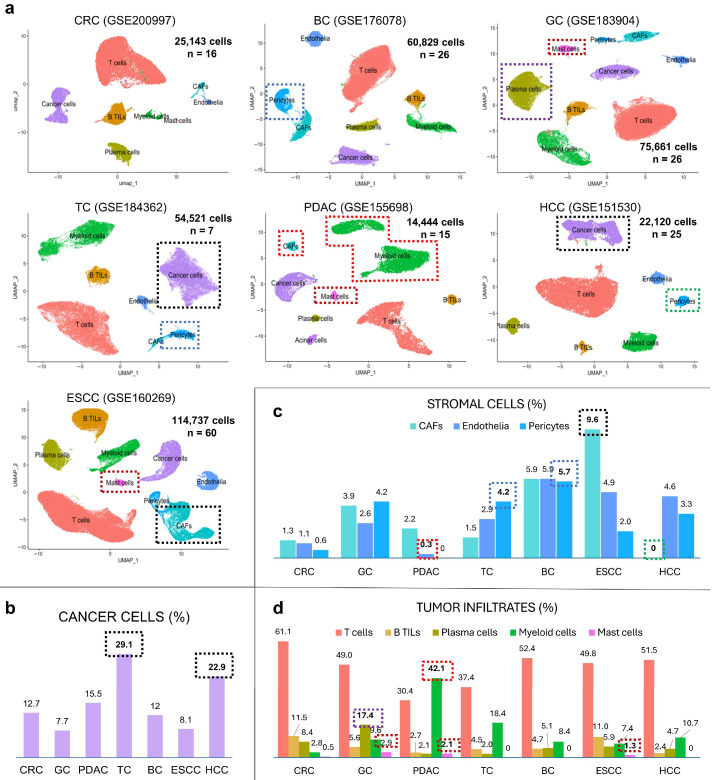
Comparative cellular composition in seven human cancers based on scRNA-seq. (**a**) UMAP plots of integrated scRNA-seq data from seven human cancers. Each dot represents a single cell colored by annotated cell type. (**b**) Relative proportion of cancer cells by cancer type. (**c**) Frequencies of stromal cells including CAFs, pericytes, and endothelial cells. (**d**) Distribution of tumor-infiltrating immune cells including T cells, B TILs, plasma cells, myeloid cells, and mast cells.

We compared cellular architectures across seven human cancers using UMAP of public scRNA-seq datasets. Dataset integration and sample details are shown in Table S1, and post-filtering cell counts are included in the UMAPs of individual cancers (Figure 1a and Figure S2). Tumor cell proportions were highest in TC and HCC, while ESCC and GC had lowest proportions (Figure 1b).

Stromal components—fibroblasts, pericytes, and endothelial cells—were quantified (Figure 1c). In CRC and PDAC, fibroblasts and pericytes formed overlapping clusters, suggesting their lineage similarity. *RGS5* confirmed pericyte identity within CAFs cluster in CRC. Due to minimal *PECAM1* expression in PDAC, alternative endothelial markers such as *ACKR1* and *RAMP2* were assessed; *RAMP2* was notably expressed in CAFs cluster, suggesting phenotypic similarity between endothelial cells and CAFs within the stromal components. PDAC showed markedly reduced endothelial contents (Figure 1c, red frames), while CAFs were the most abundant in ESCC (Figure 1c, black frame), followed by BC and GC. In contrast, BC and TC showed higher pericyte levels than CAFs (Figure 1c, blue frames). Surprisingly, no distinct CAF cluster was identified in HCC and contained *RGS5* positive “stellate cells” indicative of pericytes feature (Figure 1c, green frame).

Immune infiltrates included T cells, B TILs, plasma cells, myeloid cells, and mast cells (Figure 1d). T cells were the most abundant in CRC (61.1%) and BC (52.4%), but lower in PDAC (30.4%), suggesting immune suppression. PDAC also showed the highest proportion of myeloid cells (Figure 1d, red frame). GC uniquely showed plasma cell enrichment (Figure 1d, purple frame), reflecting diffuse type histology [9]. Mast cells appeared in PDAC, ESCC, and GC, but scarce in BC, TC, and HCC (Figure 1d, red-brown frames).

### 3.2. Discovery of CXCR2/CXCR1 Myeloid Cells Unique to PDAC

**Figure 2:**
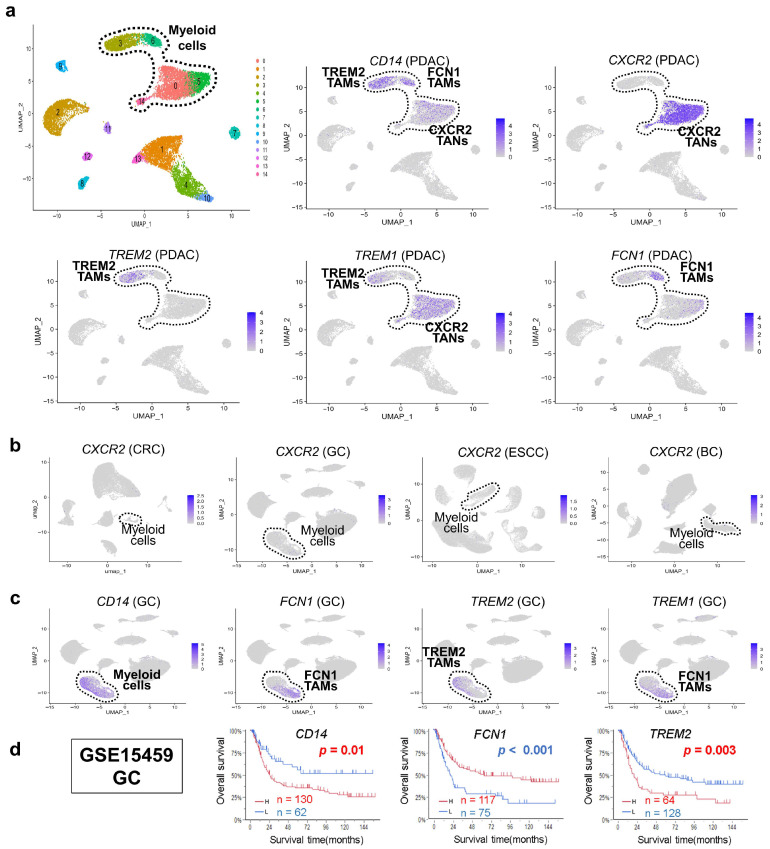
Identification of CXCR2-positive myeloid subsets unique to PDAC. (**a**) Clustering of myeloid cells in PDAC scRNA-seq data identified *CD14*-positive populations localized to clusters 0, 3, 5, 6, and 14. These were subclassified into *TREM2* and *FCN1* TAMs. Clusters 0, 5, and 14 co-expressed *CXCR2* and were reclassified as *CXCR2*-positive TANs (*CXCR2* TANs). (**b**) Comparative analysis of *CXCR2+* myeloid cells across cancers revealed a PDAC-specific enrichment. (**c**) In GC, *CXCR2* TANs were absent, and *TREM1* was confined to *FCN1* TAMs. PDAC uniquely showed overlapping *TREM1* and *TREM2* expression in *FCN1* TAMs. (**d**) Kaplan–Meier survival curves in GC (GSE15459) indicated that high expression of *CD14*, *FCN1*, and *TREM2* was associated with poor prognosis.

Given the high myeloid cell fraction in PDAC (42.1%, Figure 1d), we annotated myeloid subpopulations using canonical markers (Figure 2a). *CD14*-positive cells localized to clusters 0, 3, 5, 6, and 14 and were classified as mutually exclusive *TREM2* and *FCN1* tumor-associated macrophages (TAMs), as previously reported [17].

Clusters 0, 5, and 14 showed high *CXCR2* expression together with *CXCR1*, aligning with granulocytes [11] and designated here as *CXCR2* TANs [4]. These cells also expressed *TREM1* (Figure 2a) and were enriched in PDAC, but absent in other cancers (Figure 2b), suggesting a distinct PDAC-associated myeloid feature.

In contrast, GC showed abundant myeloid cells lacking *CXCR2* TANs. *TREM1* was restricted to *FCN1* TAMs (Figure 2c) differently from PDAC. Prognostic analysis in GC indicated that high *CD14* and *TREM2* expression correlated with poor survival (Figure 2d). Given that *TREM2* TAMs resemble CAFs-induced M2 macrophages [18], their dominance may underlie poor outcomes in GC lacking *CXCR2* TANs. We herein selected GC for survival analysis rather than PDAC because *CXCR2*-expressing myeloid cells were absent in GC, whereas in PDAC, *FCN1* expression was partially shared with *CXCR2* clusters. In addition, PDAC shows a uniformly poor prognosis, making it unsuitable for survival stratification. GC, on the other hand, exhibited a relatively high proportion of myeloid cells despite the absence of CXCR2^+^ populations, providing a more appropriate context to evaluate the prognostic significance of *TREM2* expression without overlapping signals.

### 3.3. Intercellular Interaction Analysis in PDAC

**Figure 3:**
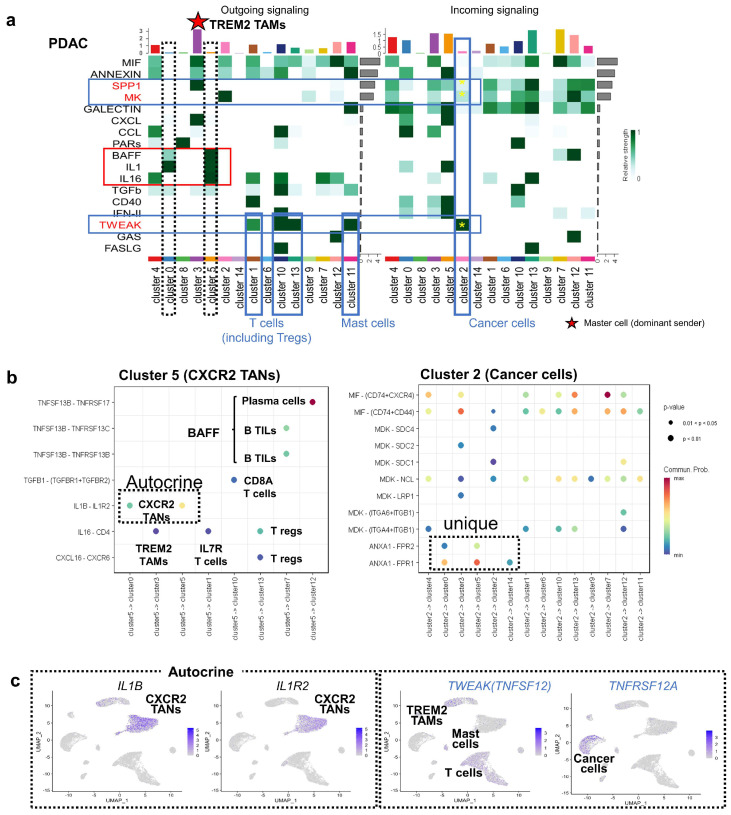
CellChat-based inference of intercellular signaling in PDAC. (**a**) Heatmap of outgoing and incoming signaling across PDAC cell clusters. *TREM2* TAMs (cluster 3) acted as dominant sender (“master”) cells, whereas *CXCR2* TANs (clusters 0 and 5) showed minimal outgoing signaling. (**b**) *CXCR2* TANs expressed *TNFSF13B*, *IL1B*, *IL16*, and *TGFB1*, contributing to paracrine signaling with T and B cells and an autocrine loop via *IL1B*–*IL1R2*. Minimal or no detectable communication was observed with cancer cells. Cluster 2 cancer cells showed unique receptor-ligand profiles. (**c**) Feature plots show *IL1B* and *IL1R2* expression in *CXCR2* TANs, and *TNFRSF12A* in cancer cells. *TWEAK* (*TNFSF12*) from immune cells may signal to tumors via *TNFRSF12A*.

We analyzed intercellular signaling in PDAC using CellChat [19]. *TREM2* TAMs (cluster 3) were the most dominant senders, whereas *CXCR2* TANs (clusters 0 and 5) exhibited minimal sender activity according to CellChat probability scores (Figure 3a, dotted frames), likely due to the absence of key signaling pathways such as *MIF*, *SPP1*, and *CXCL*, which were predominantly active in *TREM2* TAMs. Despite such low activity, *CXCR2* TANs uniquely showed outgoing signals via BAFF, IL1, IL16, and TGFB pathways (Figure 3a, red frame), forming paracrine networks with T and B cells via *BAFF* (*TNFSF13B*), *IL16*, and *TGFB1* (Figure 3b, left panel). They also exhibited autocrine *IL1B*–*IL1R2* signaling (Figure 3b,c), but lacked interactions with cancer cells (cluster 2) (Figure 3a, red frame). *TGFB1* receptors were expressed in IFNG-positive T cells (cluster 10).

PDAC cancer cells (cluster 2) expressed receptors for *SPP1*, *MDK*, and *TWEAK* (*TNFSF12*), with *TWEAK* showing the strongest interaction (Figure 3a, blue frame). *TWEAK* originated from T cells and mast cells, implicating it in tumor aggressiveness, and cancer cells clearly expressed *TNFRSF12A* (Figure 3c, right panel). Additionally, cancer cells secreted *MIF*, *MDK*, and *ANXA1* (Figure 3b, right panel). *ANXA1* was uniquely expressed in PDAC tumor cells, unlike other cancers (as described later). *ANXA1* supports an immunosuppressive TME, promoting M2 polarization represented by *TREM2* TAMs mobilization [20], ANXA1 derived from cancer cells may be involved in the formation of a TME specific to pancreatic cancer.

### 3.4. Comparative scRNA-seq Reveals Cancer-Specific Molecular Features

**Figure 4:**
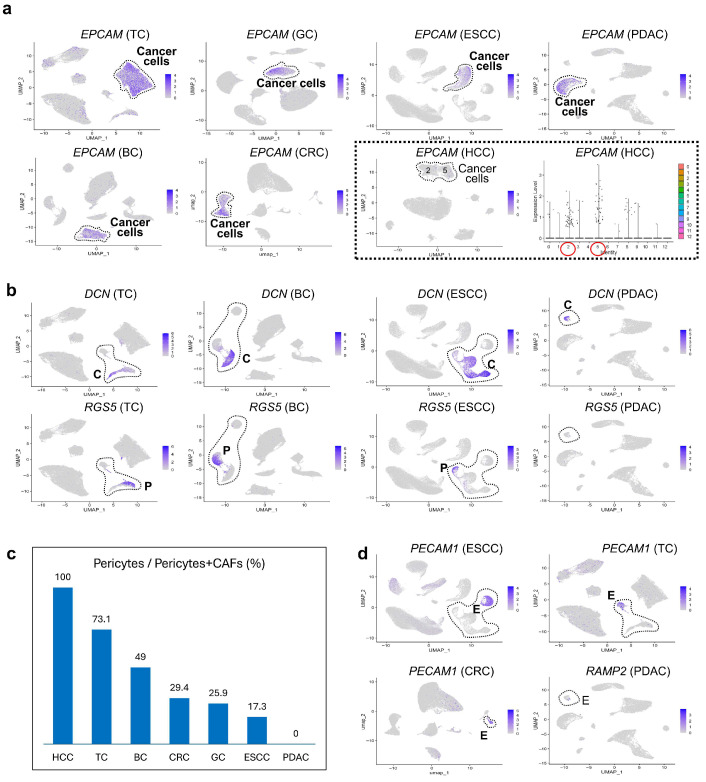
Comparative analysis of cancer- and stroma-associated features across tumor types. (**a**) Expression of EPCAM in cancer cell clusters across seven tumor types, with notably low expression in HCC. (**b**) Feature plots of stromal markers DCN (CAF marker) and RGS5 (pericyte marker) reveal heterogeneity among TC, BC, ESCC, and PDAC. (**c**) Relative pericyte abundance among stromal cells, calculated as the proportion of pericytes to the sum of pericytes and CAFs. TC and BC showed higher pericyte fractions, while PDAC had minimal content. (**d**) Feature plots of endothelial markers PECAM1 and RAMP2 show reduced endothelial signals in PDAC.

*EPCAM* is a well-known cancer-initiating gene in HCC [21]. However, comparative oncology revealed low *EPCAM* expression in HCC, unlike its strong expression in other tumor types (Figure 4a). In HCC, cluster 2 (*C1S* HCC) expressed complement-related genes such as *C1S* (Figure S3a), and cluster 5 (*CD24* HCC) showed stemness-associated markers including *CD24*. HCC was the only tumor among the seven studied that rarely metastasized to lymph nodes, a finding that may reflect differences in *EPCAM* expression among cancers.

Stromal profiling showed that TC and BC harbored a higher proportion of pericytes than CAFs (Figure 4b: P represents pericytes, while C shows CAFs). In HCC, the entire stromal population consisted of pericytes alone (no CAFs, Figure S3b), whereas in PDAC, pericytes proportion was nearly zero (Figure 4c). Although *RGS5* was weakly expressed in PDAC stromal cells, no distinct pericyte clusters were observed. High pericyte prevalence in TC and BC may correlate with favorable survival. In contrast, both pericyte and endothelial cell populations were markedly reduced in PDAC and CRC (Figure 4d). Notably, in PDAC, endothelial cells failed to form distinct clusters. Only a small fraction expressed vascular marker *RAMP2* and *ACKR1*, consistent with the hypovascular nature of PDAC.

### 3.5. Unique Characteristics of CAFs Revealed by Comparative Oncology

CAFs are generally classified into myofibroblasts (myCAFs), which promote tumor progression and immune evasion via *TREM2* TAMs [18], and inflammatory CAFs (iCAFs), which drive local inflammation response [22]. In PDAC, *COL12A1* has been reported as a myCAFs marker, whereas *COL14A1* marks iCAFs [23]. Our data showed that *DCN* and *C1S* are broadly expressed across both CAFs subtypes (Figure 4b and Figure S3a), serving as potential panCAFs markers.

Comparative oncology revealed strong *COL12A1* expression in ESCC, BC, and GC, moderate in CRC, and minimal in TC and HCC (Figure S3b), likely reflecting definite myCAFs abundance (Figure 4c). In PDAC, restriction analysis confirmed robust *COL12A1* expression in CAFs. Although both *C1S* and *DCN* dominantly mark CAFs alone, *C1S* was weakly detected in pericytes, but unexpectedly expressed in HCC cancer cells (*C1S* HCC, Figure S3a).

*CXCL12*, an alternative iCAFs marker [24], is also found in endothelial cells and thus lacks CAFs specificity (Figure S3c). Nonetheless, it remains useful for iCAFs/myCAFs distinction. High *CXCL12* expression was observed in ESCC and BC, with mutually exclusive distribution from *COL12A1*, particularly in ESCC (Figure S3c), highlighting these cancers as ideal models for CAFs subtype research.

**Figure 5:**
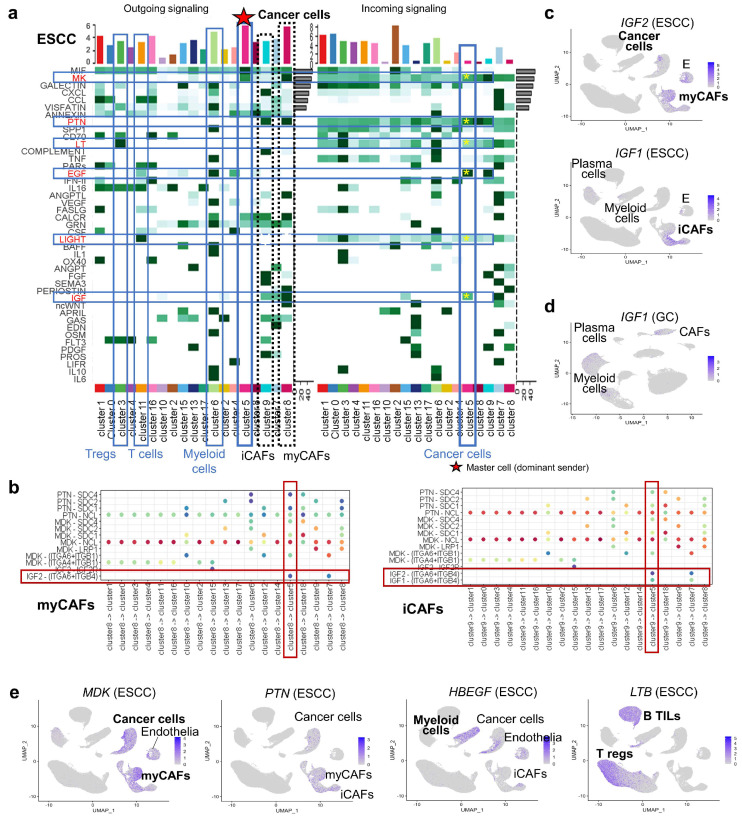
CAF subtype–derived signaling pathways in ESCC identified by CellChat analysis. (**a**) Heatmap of outgoing signaling probabilities in ESCC, highlighting cancer cells as major signal senders and diverse paracrine routes targeting other cell types. (**b**) Ligand–receptor interactions from myCAFs and iCAFs include subtype-specific signals such as *IGF2* (myCAFs) and *IGF1* (iCAFs), directed toward cancer cells. (**c**) Feature plots show *IGF1* and *IGF2* expression in ESCC stromal subtypes. (**d**) In GC, *IGF1* is expressed in plasma cells rather than CAFs. (**e**) Additional CAF-derived ligands (*MDK*, *PTN*, *HBEGF*, and *LTB*) show subtype- and cell-type-specific expression patterns.

In ESCC, CellChat analysis revealed that *IGF1* and *IGF2*, from iCAFs and myCAFs, respectively, were predominant ligands targeting tumor cells (Figure 5a–c). In GC, IGF pathway was identified from plasma cells to endothelia differently from ESCC (Figure S4a). scRNA-seq confirmed unique *IGF1* expression in plasma cells (Figure 5d).

In ESCC, additional incoming signaling patterns such as MK, PTN, LT, EGF, and LIGHT pathways were outstanding (Figure 5a, yellow asterisks), which were confirmed as distinct sender populations (*MDK* from myCAFs, *PTN* from CAFs, *HBEGF* from myeloid cells, and *LTB* from Tregs and B TILs) in scRNA-seq analysis (Figure 5e). These subtype-specific ligands highlight therapeutic targets in ESCC.

### 3.6. CellChat Reveals Distinct Sender Cell Populations Across Cancers

**Figure 6:**
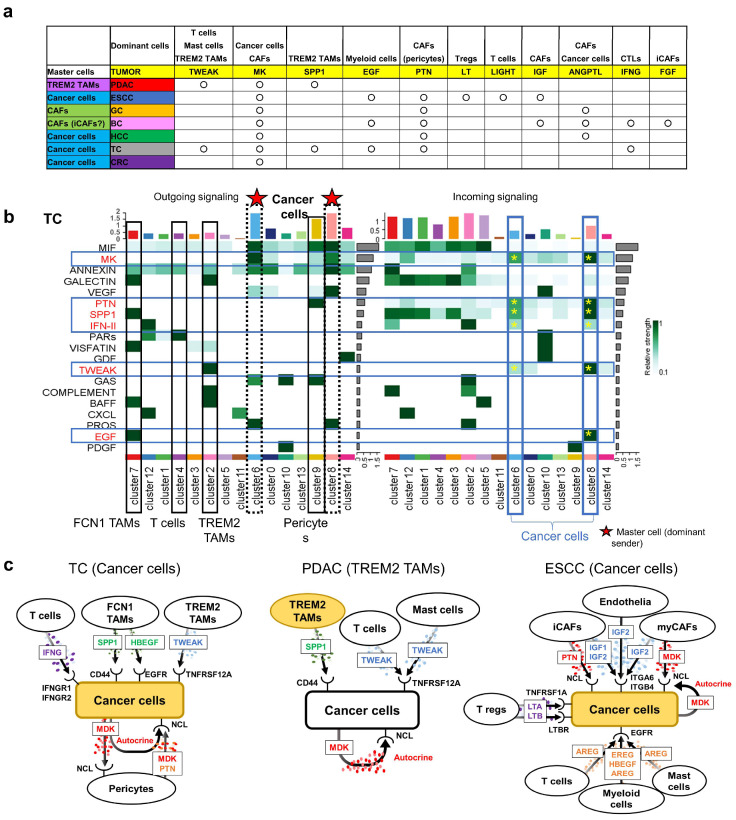
Divergent master cell populations and tumor-directed signaling across cancers. (**a**) Summary of dominant sender (“master”) clusters and signaling pathways in seven cancers. TREM2 TAMs were dominant in PDAC; CAFs in GC and BC; and tumor cells in ESCC, HCC, TC, and CRC. (**b**) Outgoing signaling heatmap in TC shows multiple tumor clusters (e.g., clusters 6 and 8) as active senders. (**c**) Diagrams of key ligand–receptor signaling toward tumor cells in TC, PDAC, and ESCC, including MDK, PTN, SPP1, TWEAK, EGF, and IFNG. Shared pathways had distinct cellular sources across tumor types.

CellChat analysis identified distinct dominant signaling senders “dominant signaling cell populations” across tumor types (Figure 6a). In PDAC, *TREM2* TAMs were the most dominant signaling cell populations (Figure 3a, red asterisks), whereas CAFs played this role in GC and BC (Figure S4a,b, red asterisks). In ESCC, TC, HCC, and CRC, cancer cells acted as dominant senders (Figure 5a, Figure 6b and Figure S4c,d, respectively, red asterisks). In TC, clusters 6 (*TPO* TC) and 8 (*CITED1* TC) showed the strongest outgoing signals (Figure 6b, red asterisks). *TPO* encodes a key enzyme for thyroid hormone synthesis, while *CITED1* is implicated in more aggressive papillary TC [25].

TC tumor cells expressed receptors for multiple signaling pathways, including MK, PTN, SPP1, TWEAK, EGF, and IFN-II (IFNG) (Figure 6b, blue frames). Notably, the IFNG pathway was unique to TC, possibly contributing to its low malignancy [26] (Figure 6c, left panel), similar with BC. Although TC and PDAC share pathways such as TWEAK, SPP1, and MK, their dominant signaling cell populations differ, suggesting divergent biological outcomes (Figure 6c, of TC, PDAC, and ESCC shown in yellow).

MK pathway activation was observed across all cancers (Figure 6a), involving both tumor cells and CAFs, while PTN and ANGPTL pathways, which share receptors with MK, were expressed dominantly in pericytes. These pathways were commonly activated in GC, BC, and HCC (Figure 6a). The EGF pathway was myeloid derived from ESCC, BC, and TC, while IGF was uniquely secreted by CAFs in ESCC and BC (Figure 6a). Unexpectedly, CRC showed only MK pathway activation (Figure S4d), suggesting a primarily cell-intrinsic malignant program.

### 3.7. Retention of Tumor-Suppressor Genes in TC Tumor Cells

We recently identified 115 cancer-associated fibroblast genes (CAFGs) with high stromal specificity (stroma-to-epithelial ratio ≥10) and strong correlation with *SPARC* expression in CRC tissue (GSE35602) [27,28]. These genes were minimally expressed in cancer cells and associated with poor prognosis in a separate colon cancer cohort (GSE17538), although this observation was limited to that dataset.

**Figure 7:**
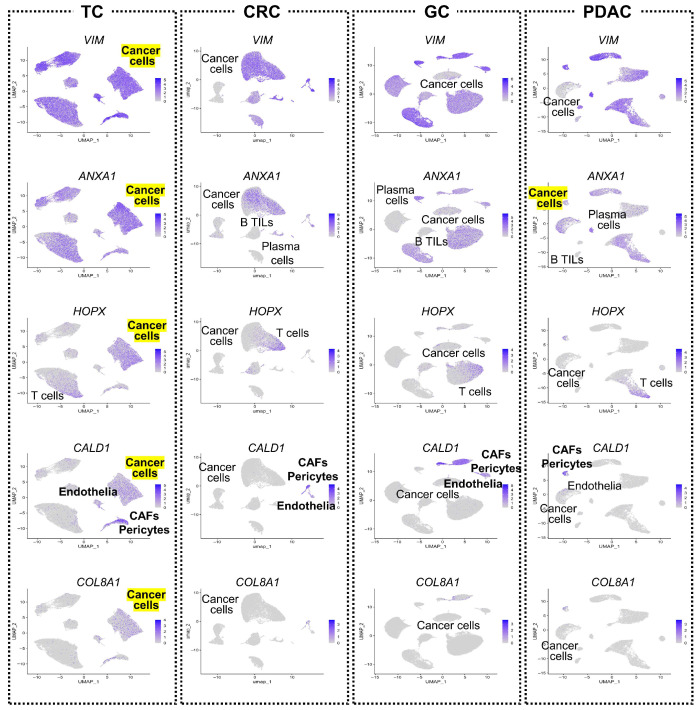
Expression of CAF-associated tumor-suppressor genes in thyroid and other cancers. Feature plots showing the expression of selected CAFGs—*VIM*, *ANXA1*, *HOPX*, *CALD1*, and *COL8A1*—in tumor cells from TC, CRC, GC, and PDAC. These genes were minimally expressed in tumor cells of CRC, GC, and PDAC, but retained in TC, particularly *VIM* and *HOPX*.

In this current study, several CAFGs—*VIM*, *ANXA1*, *HOPX*, *CALD1*, and *COL8A1*—were confirmed highly expressed in TC cells, but not in those of other cancers (Figure 7, except *ANXA1* in PDAC). Among them, *VIM* and *HOPX* are frequently silenced by promoter hypermethylation in other malignancies, and *HOPX* functions as a potent tumor suppressor [29,30]. Retention of tumor-suppressor gene expression in TC tumor cells may contribute to its distinct biology compared with more aggressive cancers.

## 4. Discussion

Comparative scRNA-seq analysis revealed distinct microenvironmental features across cancers. All data were generated using the 10x Genomics platform, ensuring a shared technical basis for single-cell transcriptomic profiling. PDAC, known for its high malignancy, exhibited a significantly higher RNA count distribution compared to other cancers (Figure S5). Therefore, if cells were selected using the same threshold, important cells may have been excluded in PDAC. To address this, we also generated a UMAP using an upper limit of 7000 transcripts (Figure S6a). However, to maintain consistency and avoid complicating the analysis, we followed Seurat’s tutorial recommendation and created UMAPs using the standard threshold of 2500 transcripts through this paper, and used those results for comparison.

One of the most prominent findings in our study was the unique molecular characteristics of PDAC. Notably, PDAC exhibited a markedly higher proportion of myeloid cells compared with other cancer types, and *CXCR2*-positive cells (designated herein as CXCR2 TANs) were almost exclusively observed in PDAC. In the original article of PDAC [11], *CXCR2*-positive cells were referred to as granulocytes and identified as the dominant myeloid subtype; they also showed synchronized expression of *CXCR1* and *IL1R2* as in our study. These consensus findings suggested an autocrine *IL1B* signaling loop involved in their activation of PDAC, mediated by TANs. In mice model, tumor cells-derived L1β promoted the activation and secretory phenotype of quiescent pancreatic stellate cells and established an immunosuppressive milieu mediated by M2 macrophages, myeloid-derived suppressor cells, CD1d^hi^CD5^+^ regulatory B cells, and Th17 cells [31].

However, in our scRNA-seq analysis, *IL1B*/*IL1R2* were scarcely expressed outside of TANs, including in cancer cells, suggesting that *IL1B*-mediated cancer progression is likely driven through TANs. Interestingly, in the mice model, antibody-mediated neutralization of IL1β significantly enhanced the antitumor activity of α-PD-1 and was accompanied by increased tumor infiltration of CD8^+^ T cells [31].

Another characteristic feature of PDAC was the scarcity of an endothelial cell cluster, with endothelial cells seemingly grouped within the CAFs population. Within the CAFs, we observed a cluster of *RAMP2*-positive cells, which we interpret as representing the endothelial component, potentially accounting for the hypovascular nature of PDAC tumors [32]. This finding is consistent with the original article [11] and our re-analysis of a UMAP using an upper limit of 7000 transcripts (Figure S6a), which also reported a very low abundance of endothelial cells in PDAC. *ACKR1*, which is known to be oncogenic in the vasculature, shares ligands (CXCLs) with *CXCR2* expressed on TANs [33,34]. In our scRNA-seq analysis, however, the expression of *ACKR1* was extremely low (actually absent). This finding suggests that the limited angiogenesis might be a result of competition with TANs. However, this hypothesis should be experimentally validated.

Cell–cell communication analysis using CellChat revealed that *TREM2* TAMs were the most dominant sender cells in PDAC, whereas *CXCR2* TANs exhibited limited outgoing signaling, mainly through the BAFF, IL1, and IL16 pathways. Interestingly, *CXCR2* TANs did not show interactions with cancer cells; their interactions were limited to other tumor-infiltrating immune cells. This observation is consistent with previous reports showing that anti-IL1B antibody treatment is effective in mouse models [31]. Among cancer cell receptors, SPP1, MK, and TWEAK were identified as potential recipients, with the *SPP1*–*CD44* axis mediating interactions with *TREM2* TAMs, consistent with recent studies [35].

The next most notable finding after those observed in PDAC was related to *EPCAM* expression in HCC. *EPCAM* has previously been reported to function as a stem cell marker or a marker for cancer-initiating cells in HCC [36,37]. However, unlike in other epithelial-derived cancers, *EPCAM* expression was not detected at levels consistent with those of a typical epithelial marker in our dataset. One possible explanation is that the HCC samples we analyzed were predominantly from core needle biopsies, but not from surgical resection differently from other cancers. While this observation requires cautious interpretation, it is worth noting that other epithelial markers such as *KRT18* showed strong expression in HCC, similar to other cancer types. Therefore, this difference in *EPCAM* expression may represent a novel insight revealed through comparative oncology. In the original study [12], HCC and cholangiocarcinoma (CCC) samples were analyzed together, which may have masked this distinction.

Interestingly, *C1S*, a typical pan-CAF marker, was strongly expressed in HCC tumor cells, suggesting an organ-specific differentiation. It is known that components of the complement system, including *C1S*, are highly expressed in normal hepatocytes [38]. In addition, our comparative analysis revealed that CAFs and pericytes could be clearly distinguished in most cancers except HCC based on *DCN* (a CAF marker) and *RGS5* (a pericyte marker). The proportion of *RGS5*-positive pericytes within fibroblast-lineage cells was relatively high in both less aggressive cancers (such as TC and BC) and low in highly aggressive ones (such as ESCC and PDAC). Further analysis of pericyte-associated signaling may help to clarify the regulatory genes underlying this heterogeneity. When examining whether the low presence of pericytes in BC is related to specific subtypes, it was found that in luminal BC, pericytes outnumber CAFs, whereas in Triple-Negative Breast Cancer (TNBC), pericytes are fewer than CAFs (described later). These findings suggest that the overall trend observed in BC reflects the characteristics of luminal BC and cannot be considered representative of TNBC.

In our clustering analysis, ESCC was the only cancer type in which CAFs were split into two distinct clusters (iCAFs and myCAFs). Notably, we identified *IGF1* and *IGF2* as being specifically expressed in each of these respective CAFs clusters, highlighting potential functional differences between them. *CXCL12*, known to be induced by *IGF2* and to promote immune suppression [39], overlapped with *IGF1* expression rather than *IGF2* in ESCC, suggesting a possible link between interCAFs IGF signaling and immunomodulation in this cancer. Collectively, these findings indicate differential roles of IGF molecules in shaping the TME across cancers. The original study of ESCC [13] subdivided CAFs into four clusters—CAFs1 (*CXCL1*^+^/*CXCL8*^−^), CAFs2 (*CXCL1*^+^/*CXCL8*^+^), and CAFs3/4 (*MMP11*^+^)—and performed trajectory analysis suggesting that myCAFs-like CAFs3/4 originated from normal fibroblasts through CAFs1, whereas another trajectory arising from *RGS5*^+^ pericytes mediated by CAFs2 and CAFs1/CAFs2 ultimately merged with myCAFs. Considering that *CXCL1* and *CXCL8* are established markers for iCAFs, these findings imply that myCAFs may arise from two distinct iCAF lineages with different origins.

In GC, CAFs could not be clearly separated into two distinct clusters. This may be attributed to the heterogeneity of GC, which comprises two major histological subtypes: diffuse type and intestinal type. When we generated separate UMAPs for each subtype and compared them (Figure S6b), we for the first time found that diffuse-type GC exhibited a markedly higher frequency of vascular endothelial cells, while the numbers of cancer cells and CAFs were significantly lower. There may be differences in treatment strategies for these GCs based on variations in angiogenesis. While the original study did not emphasize this point except unique distribution of plasma cells between the individual subtypes [9]. Further investigations into the qualitative properties of the vasculature in diffuse-type GC may provide novel insights and lead to the development of new treatment approaches. Finally, *IGF1* expression was detected in plasma cells of diffuse type GC, consistent with the original observation [9], supporting the relevance of IGF signaling within the GC microenvironment.

A similar issue of heterogeneity was observed in BC, which comprises distinct subtypes such as luminal BC, HER2 BC, and TNBC (also referred to as basal-like). To examine subtype-specific features, we newly generated separate UMAPs for each histological subtype and compared their cellular compositions (Figure S6c). Luminal BC showed a higher proportion of cancer cells, as well as elevated levels of pericytes and vascular endothelial cells. In contrast, TNBC was enriched with plasma cells, B TILs, and myeloid cells, suggesting that immune infiltration—particularly involving immunosuppressive myeloid populations—may contribute to the aggressiveness of this subtype. HER2-enriched BC displayed a higher proportion of T cells. Our findings were consistent with those reported in the original dataset [8]. Notably, the limited efficacy of immune checkpoint inhibitors (ICIs) in HER2-positive BC is well documented [40], and our results highlight the importance of investigating qualitative differences in T cell populations within this subtype, which may provide new insights into its tumor biology. Overall, in BC, understanding the subtype-specific characteristics of the TME appears to be crucial for elucidating disease mechanisms.

In papillary TC, several mesenchymal markers, including *VIM*, were found to be strongly expressed in cancer cells. For example, *HOPX*, a gene known for its tumor-suppressor functions, including in TC [41], showed minimal expression in cancer cells of other tumor types, but was highly expressed in papillary TC cells. The original study reported that *HOPX* expression was elevated in the radioactive iodine (RAI) refractory distant metastasis of TC [10], reaffirming the dual nature of this gene. These findings suggest that *HOPX* expression in cancer cells with mesenchymal characteristics may reflect a context-dependent role rather than a purely tumor-suppressive function.

Common signaling patterns were observed across multiple cancer types. For instance, both PDAC and TC shared activation of the TWEAK and SPP1 pathways on cancer cells despite their opposing malignancy, suggesting that cancer aggressiveness may depend more on the immune and stromal context—such as *CXCR2* TAN enrichment or pericyte abundance—than on individual oncogenic signaling events. Integrating these immune and stromal factors may therefore provide a more accurate framework for understanding tumor behavior and for developing TME-targeted therapies.

## 5. Limitations

This study has several limitations. Although all datasets were generated using the 10x Genomics platform, they originated from different research groups and may have differed in sample preparation, tissue dissociation methods, and sequencing depth. These technical variables may have introduced inter-study variability that could influence cell composition or gene expression profiles. In addition, the relatively small number of representative cases per cancer type and the reliance on scRNA-seq alone limit the generalizability of our findings and restrict the ability to draw functional conclusions. Certain signaling pathways not covered in the current version of CellChat may also have been missing. Survival analyses were conducted using bulk transcriptomic datasets, which reflect average gene expression across all cell types in a sample. As such, these analyses may not directly link clinical outcomes to specific cell subpopulations identified by scRNA-seq, such as *TREM2*-positive TAMs. Rather, they demonstrate correlations that should be interpreted with caution.

In addition, the quality-control (QC) filtering thresholds used in this study were intentionally conservative to ensure methodological consistency across all cancer types and to minimize potential doublets that might otherwise confound downstream clustering analyses. We applied a uniform upper cutoff of 2500 detected genes (nFeature_RNA) for cell inclusion, following the official Seurat tutorial (200–2500 range), which is widely adopted in single-cell RNA-seq analyses. This approach was chosen to maintain analytical comparability in the context of comparative oncology. Examination of the nFeature_RNA distributions across cancer types (Figure S5) revealed that this upper limit excluded a substantial number of cells in certain datasets. In particular, PDAC showed globally higher gene counts, BC displayed elevated values in several case subsets, and ESCC exhibited such patterns mainly within the CD45^−^ fraction. These tendencies indicate that the applied threshold may have been overly stringent for some cancer types.

To assess the impact of this filtering criterion, we reanalyzed the PDAC dataset using a relaxed upper cutoff of 7000 genes (Figure S6a). Under this condition, pericyte and endothelial clusters became more clearly defined, and the relative proportions of CAFs, cancer cells, and pericytes increased, whereas T cells remained low in absolute numbers and thus decreased proportionally. This finding supports the interpretation that T-cell scarcity represents an inherent biological feature of PDAC rather than a filtering artifact. Nevertheless, because cells with exceptionally high transcriptional activity—such as CAFs or proliferating cancer cells—often exceed the 2500-gene threshold, it is possible that part of these transcriptionally active populations was underrepresented in our dataset. While this potential bias does not affect the qualitative trends observed across cancers, it should be recognized as a limitation inherent to stringent filtering.

Overall, although the stricter thresholds reduced total cell counts, they provided a standardized and conservative framework for cross-cancer comparison and did not alter the principal cellular hierarchies or signaling patterns identified in this study. Importantly, even under these uniform analytical conditions, PDAC consistently displayed a markedly low abundance of endothelial populations, reinforcing its known hypovascular phenotype as a biologically robust feature.

Furthermore, several biological interpretations in this study should be considered as hypotheses rather than conclusions. For example, the proposed link between low *EPCAM* expression and the absence of lymph node metastasis in HCC, and the suggestion that *CXCR2*-mediated ligand competition underlies the hypovascularity of pancreatic cancer, are intriguing, but not directly demonstrated by the present data. These mechanistic assumptions remain speculative and require further validation.

## 6. Conclusions

In summary, this comparative scRNA-seq study across seven human cancers reveals both shared and cancer-specific tumor phenotypes and intercellular signaling networks. Key findings include the enrichment of immunosuppressive CXCR2^+^ TANs and reduced vascular components in PDAC, diverse CAF subsets with distinct signaling roles in ESCC and BC, unique plasma-cell features in GC, and organ-specific stromal adaptations such as low *EPCAM* expression in HCC and preserved tumor suppressor and/or mesenchymal genes in TC. These observations advance our understanding of the immune–stromal architecture driving cancer behavior and highlight the potential of cross-cancer single-cell approaches to uncover new therapeutic targets in surgical oncology.

## Data Availability

The scRNA-seq datasets analyzed in this study are publicly available from the GEO under the accession numbers listed in the Methods section. Processed data and analysis scripts used for figure generation and pathway analysis are available from the corresponding author upon reasonable request.

## Supplementary Materials

The following supporting information can be downloaded at: https://www.mdpi.com/article/10.3390/cancers17213527/s1, Figure S1: Overview of the analytical workflow; Figure S2: Unannotated Cluster Numbering in UMAPs of Six Human Cancers; Figure S3: Expression of CAF-Associated Markers Across Cancer Types; Figure S4: Identification of Dominant Sender Populations and Tumor-Directed Pathways in Four Human Cancers; Figure S5: Distribution of detected gene counts (nFeature_RNA) across seven cancer types analyzed in this study; Figure S6: Cellular composition in PDAC and subtype-specific features in GC and BC; Table S1: Summary of patient cohorts and clinical characteristics of single-cell RNA-seq datasets; Table S2: Summary of the GC cohort (GSE15459) used for survival analysis; Table S3: Summary of Representative Samples Used for scRNA-seq Analysis in Each Cancer Type.

## Abbreviations

The following abbreviations are used in this manuscript:

BC	Breast cancer
B TILs	B-type tumor-infiltrating lymphocytes.
CAFs	Cancer-associated fibroblast
CAFGs	Cancer-associated fibroblast genes
CRC	Colorectal cancer
ESCC	Esophageal squamous cell carcinoma
GC	Gastric cancer
HCC	Hepatocellular carcinoma
PDAC	Pancreatic ductal adenocarcinoma
scRNA-seq	Single-cell RNA sequencing
TAN	Tumor-associated neutrophil
TC	Thyroid cancer
TME	Tumor microenvironment
TNBC	Triple-Negative Breast Cancer
UMAP	Uniform manifold approximation and projection

## References

[1] Seferbekova Z., Lomakin A., Yates L.R., Gerstung M. (2023). Spatial biology of cancer evolution. Nat. Rev. Genet..

[2] Li Y., Cai L., Wang H., Wu P., Gu W., Chen Y., Hao H., Tang K., Yi P., Liu M. (2011). Pleiotropic regulation of macrophage polarization and tumorigenesis by formyl peptide receptor-2. Oncogene.

[3] Lee H.O., Hong Y., Etlioglu H.E., Cho Y.B., Pomella V., Van den Bosch B., Vanhecke J., Verbandt S., Hong H., Min J.W. (2020). Lineage-dependent gene expression programs influence the immune landscape of colorectal cancer. Nat. Genet..

[4] Xie Y., Zhou T., Li X., Zhao K., Bai W., Hou X., Liu Z., Ni B., Zhang Z., Yan J. (2024). Targeting ESE3/EHF With Nifurtimox Inhibits CXCR2(+) Neutrophil Infiltration and Overcomes Pancreatic Cancer Resistance to Chemotherapy and Immunotherapy. Gastroenterology.

[5] Coulouarn C., Corlu A., Glaise D., Guénon I., Thorgeirsson S.S., Clément B. (2012). Hepatocyte-stellate cell cross-talk in the liver engenders a permissive inflammatory microenvironment that drives progression in hepatocellular carcinoma. Cancer Res..

[6] Croizer H., Mhaidly R., Kieffer Y., Gentric G., Djerroudi L., Leclere R., Pelon F., Robley C., Bohec M., Meng A. (2024). Deciphering the spatial landscape and plasticity of immunosuppressive fibroblasts in breast cancer. Nat. Commun..

[7] Khaliq A.M., Erdogan C., Kurt Z., Turgut S.S., Grunvald M.W., Rand T., Khare S., Borgia J.A., Hayden D.M., Pappas S.G. (2022). Refining colorectal cancer classification and clinical stratification through a single-cell atlas. Genome Biol..

[8] Wu S.Z., Al-Eryani G., Roden D.L., Junankar S., Harvey K., Andersson A., Thennavan A., Wang C., Torpy J.R., Bartonicek N. (2021). A single-cell and spatially resolved atlas of human breast cancers. Nat. Genet..

[9] Kumar V., Ramnarayanan K., Sundar R., Padmanabhan N., Srivastava S., Koiwa M., Yasuda T., Koh V., Huang K.K., Tay S.T. (2022). Single-Cell Atlas of Lineage States, Tumor Microenvironment, and Subtype-Specific Expression Programs in Gastric Cancer. Cancer Discov..

[10] Pu W., Shi X., Yu P., Zhang M., Liu Z., Tan L., Han P., Wang Y., Ji D., Gan H. (2021). Single-cell transcriptomic analysis of the tumor ecosystems underlying initiation and progression of papillary thyroid carcinoma. Nat. Commun..

[11] Steele N.G., Carpenter E.S., Kemp S.B., Sirihorachai V.R., The S., Delrosario L., Lazarus J., Amir E.D., Gunchick V., Espinoza C. (2020). Multimodal Mapping of the Tumor and Peripheral Blood Immune Landscape in Human Pancreatic Cancer. Nat. Cancer..

[12] Ma L., Wang L., Khatib S.A., Chang C.W., Heinrich S., Dominguez D.A., Forgues M., Candia J., Hernandez M.O., Kelly M. (2021). Single-cell atlas of tumor cell evolution in response to therapy in hepatocellular carcinoma and intrahepatic cholangiocarcinoma. J. Hepatol..

[13] Zhang X., Peng L., Luo Y., Zhang S., Pu Y., Chen Y., Guo W., Yao J., Shao M., Fan W. (2021). Dissecting esophageal squamous-cell carcinoma ecosystem by single-cell transcriptomic analysis. Nat. Commun..

[14] McGinnis C.S., Murrow L.M., Gartner Z.J. (2019). DoubletFinder: Doublet Detection in Single-Cell RNA Sequencing Data Using Artificial Nearest Neighbors. Cell Syst..

[15] Jin S., Guerrero-Juarez C.F., Zhang L., Chang I., Ramos R., Kuan C.H., Myung P., Plikus M.V., Nie Q. (2021). Inference and analysis of cell-cell communication using CellChat. Nat. Commun..

[16] Ooi C.H., Ivanova T., Wu J., Lee M., Tan I.B., Tao J., Ward L., Koo J.H., Gopalakrishnan V., Zhu Y. (2009). Oncogenic pathway combinations predict clinical prognosis in gastric cancer. PLoS Genet..

[17] Hornburg M., Desbois M., Lu S., Guan Y., Lo A.A., Kaufman S., Elrod A., Lotstein A., DesRochers T.M., Munoz-Rodriguez J.L. (2021). Single-cell dissection of cellular components and interactions shaping the tumor immune phenotypes in ovarian cancer. Cancer Cell..

[18] Cao Q., Sun D., Tu C., Wang J., Fu R., Gong R., Xiao Y., Liu Q., Li X. (2025). Defining gastric cancer ecology: The crucial roles of TREM2(+) macrophages and fibroblasts in tumor microenvironments. Commun. Biol..

[19] Mayer S., Milo T., Isaacson A., Halperin C., Miyara S., Stein Y., Lior C. (2023). The tumor microenvironment shows a hierarchy of cell-cell interactions dominated by fibroblasts. Nat. Commun..

[20] Li H., Courtois E.T., Sengupta D., Tan Y., Chen K.H., Goh J.J.L., Kong S.L., Chua C., Hon L.K., Tan W.S. (2017). Reference component analysis of single-cell transcriptomes elucidates cellular heterogeneity in human colorectal tumors. Nat. Genet..

[21] Yamashita T., Budhu A., Forgues M., Wang X.W. (2007). Activation of hepatic stem cell marker EpCAM by Wnt-beta-catenin signaling in hepatocellular carcinoma. Cancer Res..

[22] Arpinati L., Carradori G., Scherz-Shouval R. (2024). CAF-induced physical constraints controlling T cell state and localization in solid tumours. Nat. Rev. Cancer.

[23] Thorlacius-Ussing J., Jensen C., Nissen N.I., Cox T.R., Kalluri R., Karsdal M., Willumsen N. (2024). The collagen landscape in cancer: Profiling collagens in tumors and in circulation reveals novel markers of cancer-associated fibroblast subtypes. J. Pathol..

[24] Elyada E., Bolisetty M., Laise P., Flynn W.F., Courtois E.T., Burkhart R.A., Teinor J.A., Belleau P., Biffi G., Lucito M.S. (2019). Cross-Species Single-Cell Analysis of Pancreatic Ductal Adenocarcinoma Reveals Antigen-Presenting Cancer-Associated Fibroblasts. Cancer Discov..

[25] Aldred M.A., Huang Y., Liyanarachchi S., Pellegata N.S., Gimm O., Jhiang S., Davuluri R.V., de la Chapelle A., Eng C. (2004). Papillary and follicular thyroid carcinomas show distinctly different microarray expression profiles and can be distinguished by a minimum of five genes. J. Clin. Oncol..

[26] Wang Q.S., Shen S.Q., Sun H.W., Xing Z.X., Yang H.L. (2018). Interferon-gamma induces autophagy-associated apoptosis through induction of cPLA2-dependent mitochondrial ROS generation in colorectal cancer cells. Biochem. Biophys. Res. Commun..

[27] Okuno K., Ikemura K., Okamoto R., Oki K., Watanabe A., Kuroda Y., Kidachi M., Fujino S., Nie Y., Higuchi T. (2024). CAF-associated genes putatively representing distinct prognosis by in silico landscape of stromal components of colon cancer. PLoS ONE.

[28] Nie Y., Fujiyama Y., Shibaki S., Okamoto R., Okuno K., Oki K., Watanabe A., Kuroda Y., Goto T., Yokota K. (2025). SPARC Induces COL1A1/COL3A1 Expressions Representing Aggressive Molecular Cancer-Associated Fibroblasts Signatures and CSF1-Mediated Cancer Invasion in Colorectal Cancer. Ann. Surg. Oncol..

[29] Ren X., Yang X., Cheng B., Chen X., Zhang T., He Q., Li B., Li Y., Tang X., Wen X. (2017). HOPX hypermethylation promotes metastasis via activating SNAIL transcription in nasopharyngeal carcinoma. Nat. Commun..

[30] Cheung W.K., Zhao M., Liu Z., Stevens L.E., Cao P.D., Fang J.E., Westbrook T.F., Nguyen D.X. (2013). Control of alveolar differentiation by the lineage transcription factors GATA6 and HOPX inhibits lung adenocarcinoma metastasis. Cancer Cell.

[31] Das S., Shapiro B., Vucic E.A., Vogt S., Bar-Sagi D. (2020). Tumor Cell-Derived IL1β Promotes Desmoplasia and Immune Suppression in Pancreatic Cancer. Cancer Res..

[32] Ishigami K., Yoshimitsu K., Irie H., Shinozaki K., Nagata S., Yamaguchi K., Honda H. (2008). Imaging of intraductal tubular tumors of the pancreas. AJR Am. J. Roentgenol..

[33] Xu L., Ashkenazi A., Chaudhuri A. (2007). Duffy antigen/receptor for chemokines (DARC) attenuates angiogenesis by causing senescence in endothelial cells. Angiogenesis.

[34] Devi S., Wang Y., Chew W.K., Lima R., N A.G., Mattar C.N., Chong S.Z., Schlitzer A., Bakocevic N., Chew S. (2013). Neutrophil mobilization via plerixafor-mediated CXCR4 inhibition arises from lung demargination and blockade of neutrophil homing to the bone marrow. J. Exp. Med..

[35] Fabre T., Barron A.M.S., Christensen S.M., Asano S., Bound K., Lech M.P., Wadsworth M.H., Chen X., Wang C., Wang J. (2023). Identification of a broadly fibrogenic macrophage subset induced by type 3 inflammation. Sci. Immunol..

[36] Murakata A., Tanaka S., Mogushi K., Yasen M., Noguchi N., Irie T., Kudo A., Nakamura N., Tanaka H., Arii S. (2011). Gene expression signature of the gross morphology in hepatocellular carcinoma. Ann. Surg..

[37] Ogawa K., Tanaka S., Matsumura S., Murakata A., Ban D., Ochiai T., Irie T., Kudo A., Nakamura N., Tanabe M. (2014). EpCAM-targeted therapy for human hepatocellular carcinoma. Ann. Surg. Oncol..

[38] Ramadori G., Heinz H.P., Martin H., Meyer zum Büschenfelde K.H., Loos M. (1986). Biosynthesis of the subcomponents C1q, C1r and C1s of the first component of complement (C1) by guinea pig hepatocyte primary cultures. Eur. J. Immunol..

[39] Song D., Wu Y., Li J., Liu J., Yi Z., Wang X., Sun J., Li L., Wu Q., Chen Y. (2024). Insulin-like growth factor 2 drives fibroblast-mediated tumor immunoevasion and confers resistance to immunotherapy. J. Clin. Investig..

[40] Duesberg M., LeVee A., Chang H., Tsai K., Crossman B., Tadi M., Xu S., Wheeler D., Kang I. (2025). Breast Cancer Immunotherapy: A Team Science Approach. Cancer Treat. Res..

[41] Ooizumi Y., Katoh H., Yokota M., Watanabe M., Yamashita K. (2019). Epigenetic silencing of HOPX is critically involved in aggressive phenotypes and patient prognosis in papillary thyroid cancer. Oncotarget.

