# Supplementary material for: Comparative Oncology: Cross-Sectional Single-Cell Transcriptomic Profiling of the Tumor Microenvironment Across Seven Human Cancers
Source: Cancers (Basel). 2025 Oct 31;17(21):3527. doi: 10.3390/cancers17213527 (PMC12609998; doi:10.3390/cancers17213527)
Supplement: Supplementary file 1 [file cancers-17-03527-s001.zip › cancers-3897416-supplementary.pdf]

Supplementary Figures/Legends

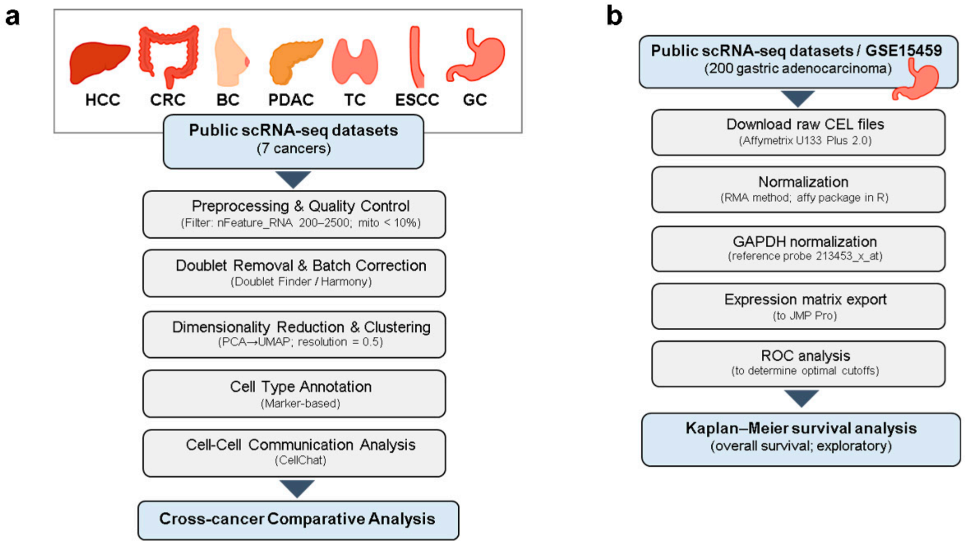

Figure S1. Overview of the analytical workflow.

(**a**) Workflow for single-cell transcriptomic analysis across seven cancer types. (**b**) Workflow for survival analysis using the GC dataset (GSE15459).

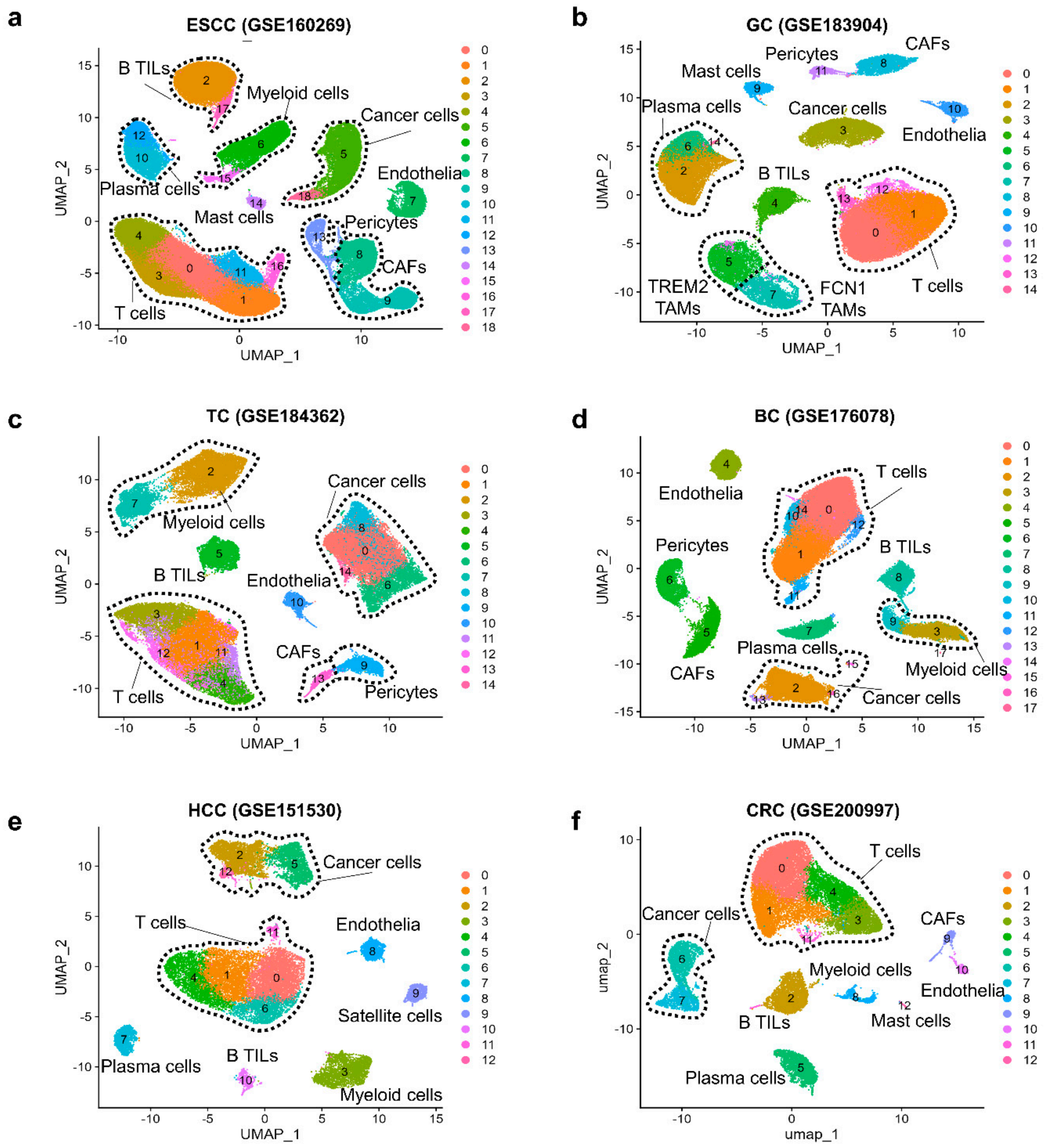

Figure S2. Unannotated Cluster Numbering in UMAPs of Six Human Cancers.

UMAP plots showing unannotated cluster numbers for ESCC (**a**), TC (**b**), GC (**c**), BC (**d**), HCC (**e**), and CRC (**f**). These cluster numbers correspond to those used in subsequent CellChat analyses. PDAC is shown separately in Figure 3.

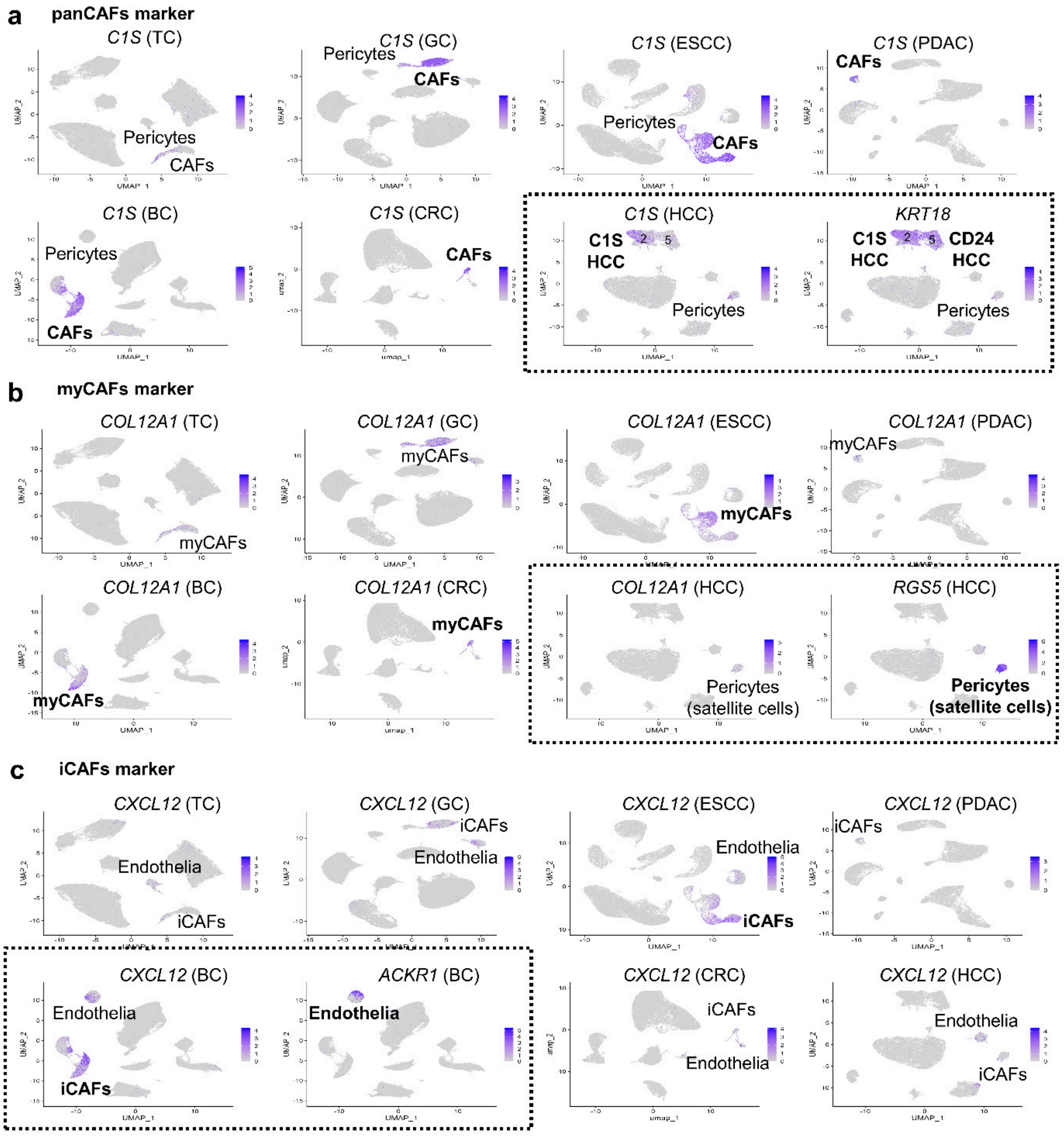

Figure S3. Expression of CAF-Associated Markers Across Cancer Types.

(**a**) Expression of *C1S*, a panCAF marker, broadly present in CAFs and weakly detected in pericytes; aberrant expression in HCC tumor cells suggests stromal gene repurposing. (**b**) Expression of *COL12A1*, a marker of myofibroblasts (myCAFs), showing high levels in ESCC, BC, and GC, and minimal expression in TC and HCC. (**c**) Expression of *CXCL12*, a marker of inflammatory CAFs (iCAFs), showing spatial separation from *COL12A1*, particularly in ESCC and BC.

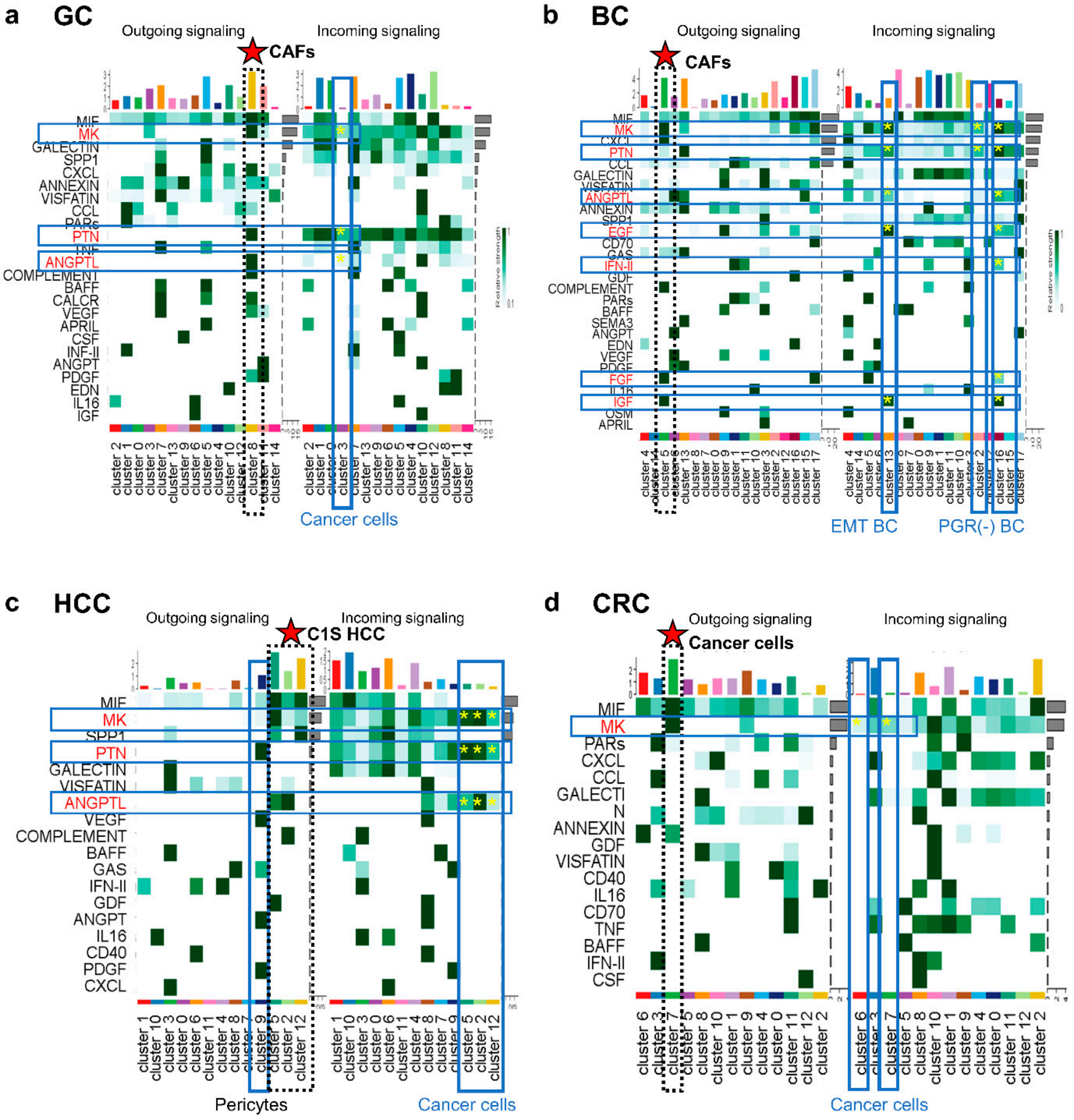

Figure S4. Identification of Dominant Sender Populations and Tumor-Directed Pathways in Four Human Cancers.

CellChat-inferred heatmaps showing outgoing signaling strength and receiver diversity across clusters in GC (**a**), BC (**b**), HCC (**c**), and CRC (**d**). Asterisks (★) mark dominant sender clusters ("dominant signaling cell populations"). Pathways with cancer cell–predominant targets highlight subtype-specific paracrine or autocrine programs.

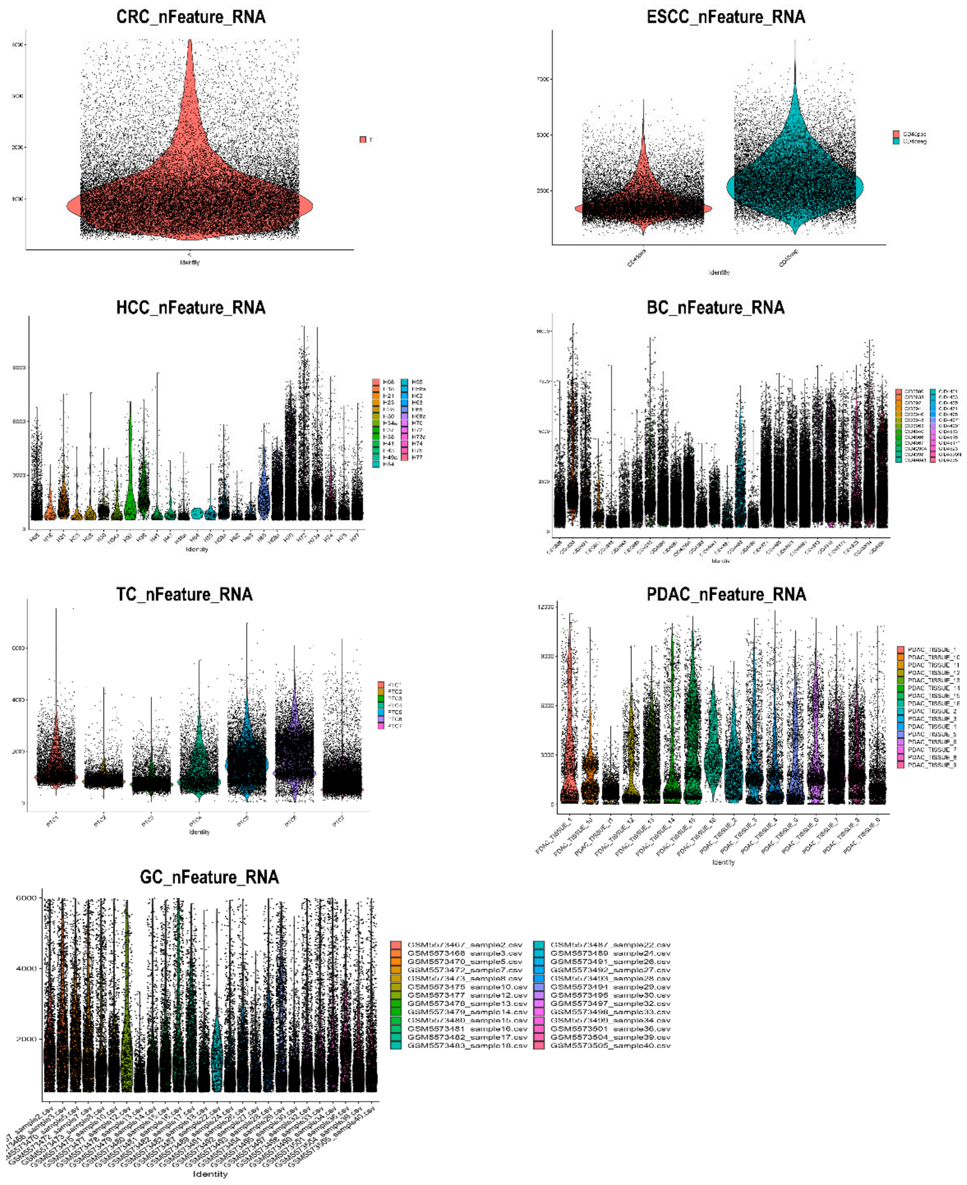

Figure S5. Distribution of detected gene counts (nFeature_RNA) across seven cancer types analyzed in this study.

Each violin plot represents the per-cell gene count distribution before quality-control filtering.

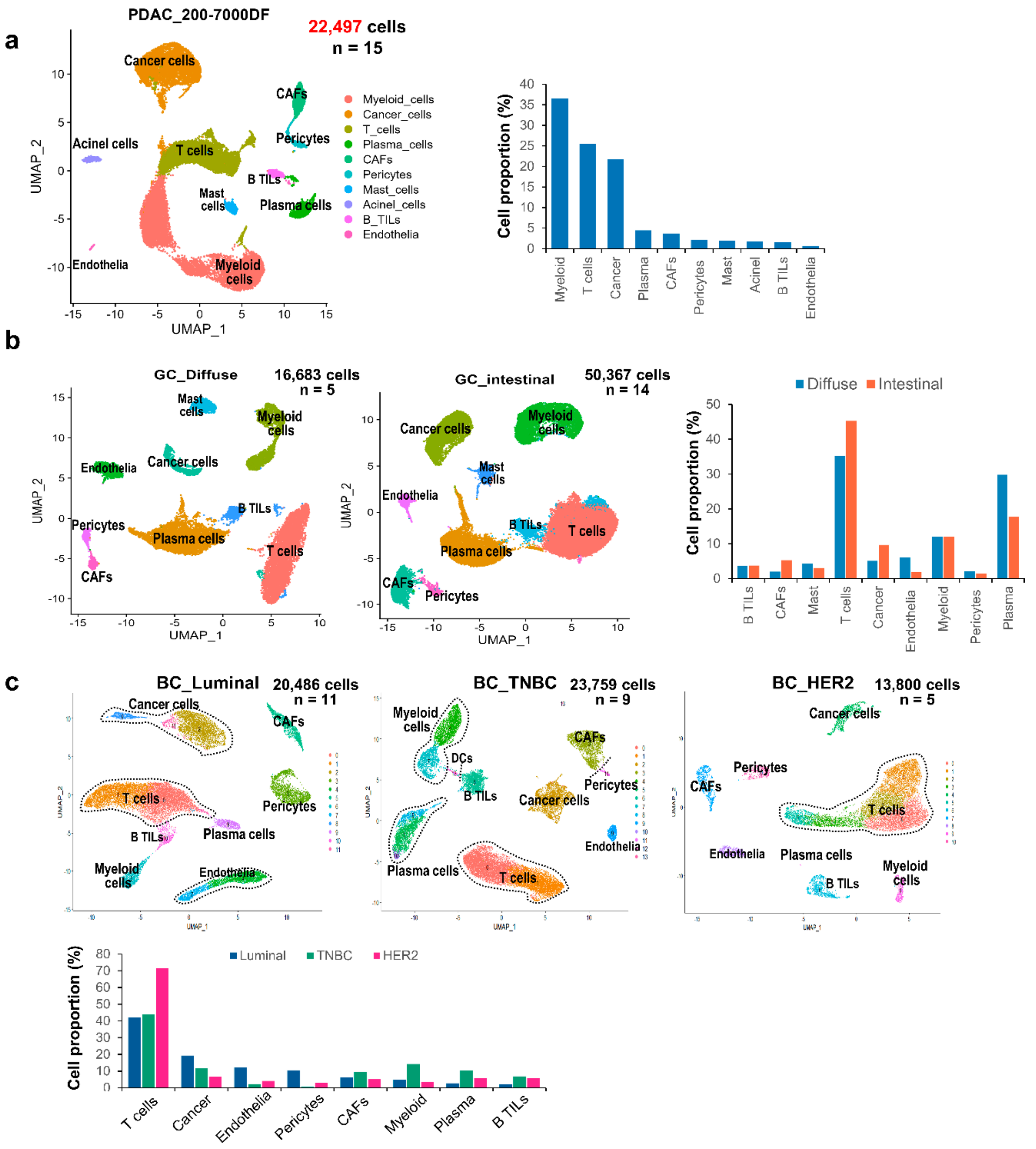

Figure S6. Cellular composition in PDAC and subtype-specific features in GC and BC.

(**a**) UMAP and cell proportion of PDAC generated with an upper filtering threshold of 7000 genes. (**b**) UMAPs and cell proportions of diffuse- and intestinal-type GC (filtering threshold: 2500 genes). (**c**) UMAPs and cell proportions of luminal, TNBC, and HER2-enriched BC (filtering threshold: 2500 genes).

Supplementary Table

Table S1. Summary of patient cohorts and clinical characteristics of single-cell RNA-seq datasets.

**Cancer type**	**GEO accession**	**n (cases)**	**Tissue type**	**Histological subtype**	**Clinical stage**	**Reference**
CRC	GSE200997	16 patients	Primary resected tumor (treatment-naïve)	Predominantly moderately differentiated adenocarcinoma; one poorly differentiated case	Stage I (n = 5), II (n = 3), III (n = 7), IV (n = 1)	Khaliq AM *et al.*, *Genome Biology*, 2022
BC	GSE176078	26 patients	Primary tumor (pre-treatment; some received neoadjuvant chemotherapy)	Estrogen receptor–positive (n = 11), HER2-positive (n = 5), triple-negative breast cancer (TNBC, n = 10)	Stage 0 (n = 1), I (n = 3), II (n = 13), III (n = 6), IV (n = 1)	Wu SZ *et al.*, *Nature Genetics*, 2021
GC	GSE183904	26 patients	Primary resected gastric adenocarcinoma	Diffuse type, intestinal type, and mixed intestinal type	Stage I (n = 3), II (n = 6), III (n = 14), IV (n = 3)	Kumar V *et al.*, *Cancer Discovery*, 2022
TC	GSE184362	7 patients	Primary papillary thyroid carcinoma (post-initial treatment)	Papillary thyroid carcinoma (PTC)	Stage I–II (age not specified in dataset)	Pu W *et al.*, *Nature Communications*, 2021
PDAC	GSE155698	16 patients (15 valid samples)	Surgical resection (n = 6) and fine-needle biopsy specimens (n = 10)	Pancreatic ductal adenocarcinoma	T1N1 (n = 2), T2N0 (n = 2), T2N1 (n = 2)	Steele NG *et al.*, *Nature Cancer*, 2020
HCC	GSE151530	25 patients	Primary hepatocellular carcinoma (treatment-naïve)	HCV-positive (n = 14), HBV-positive (n = 4), fatty liver (n = 2), HBV/HDV co-infection (n = 1)	Stage I (n = 2), II (n = 3), III (n = 3), IV (n = 17)	Ma L *et al.*, *Journal of Hepatology*, 2021
ESCC	GSE160269	60 patients	Surgically resected primary esophageal squamous cell carcinoma	Esophageal squamous cell carcinoma (ESCC)	Stage I (n = 16), II (n = 18), III (n = 26), IV (n = 0)	Zhang X *et al.*, *Nature Communications*, 2021

Publicly available scRNA-seq datasets were obtained from the Gene Expression Omnibus (GEO). All samples represent primary tumor specimens collected at diagnosis or surgical resection.

Table S2. Summary of the gastric cancer cohort (GSE15459) used for survival analysis.

Dataset	GEO accession	n (patients)	Histological subtype	Clinical stage	Platform	Outcome	Median OS (months)	Reference
GC	GSE15459	200 patients (clinical data available for 192 cases)	Diffuse (n = 75), Intestinal (n = 99), Mixed intestinal (n = 18)	Stage I (n = 31), II (n = 29), III (n = 71), IV (n = 60)	Affymetrix Human Genome U133 Plus 2.0 Array	Overall survival (1 = death)	19.7	Ooi CH *et al.*, *PLoS Genetics*, 2009

Table S3. Summary of Representative Samples Used for Single-Cell RNA Sequencing Analysis in Each Cancer Type

Cancer Type	GSE Accession	Sample ID	Cell Count	Median UMI per Cell	Notes
CRC	GSE200997	-	25143	2739	Pre-integrated
BC	GSE2176078	CID3586	5327	2064	Individually merged
GC	GSE183904	GSM5573480_sample15.csv	8822	3191.5	Individually merged
TC	GSE184362	PTC7	19733	1042	Individually merged
PDAC	GSE155698	PDAC_TISSUE_9	9182	2267	Individually merged
HCC	GSE151530	H70	3582	3181.5	Individually merged
ESCC	GSE160269	CD45pos	86573	5028	CD45+ and CD45– merged

Supplementary Methods

This section provides detailed methodological information complementary to the brief descriptions presented in the main text.

Single-cell RNA-seq Datasets and Preprocessing

Single-cell RNA sequencing (scRNA-seq) data from seven human cancers — pancreatic ductal adenocarcinoma (PDAC), hepatocellular carcinoma (HCC), esophageal squamous cell carcinoma (ESCC), breast cancer (BC), thyroid cancer, gastric cancer, and colorectal cancer (CRC) — were analyzed. Publicly available datasets were obtained from the Gene Expression Omnibus under the following accession numbers: CRC (GSE200997), BC (GSE176078), GC (GSE183904), TC (GSE184362), PDAC (GSE155698), HCC (GSE151530), and ESCC (GSE160269). For each cancer type, only tumor samples were selected; non-malignant or adjacent normal tissues were excluded based on the provided annotations.

To facilitate downstream analyses, data files were organized and preprocessed locally prior to being loaded into R (version 4.4.2) and Seurat (version 4.3.0). For CRC, a pre-integrated expression matrix from 16 tumor samples was used. For BC, GC, TC, PDAC, and HCC, scRNA-seq data were downloaded as case-specific matrices and integrated manually. For ESCC, the data were originally separated by CD45 status (CD45+ and CD45– cells) in the source dataset, and these subsets were analyzed together.

Seurat objects were constructed using the CreateSeuratObject() function with a minimum of 3 cells per gene and 200 detected features per cell, unless otherwise noted. Depending on the dataset format, either Read10X(), ReadMtx(), or read.csv() was used to import count matrices. Further quality control, including mitochondrial gene filtering, doublet removal, and batch effect correction, is described in subsequent sections.

Quality Control and Filtering

Initial quality control was performed using Seurat. Cells were retained if they expressed between 200 and 2,500 unique molecular identifiers (UMIs), as cells with UMI counts above 2,500 were considered to be potential doublets and therefore excluded. Additionally, cells with more than 10% of transcripts derived from mitochondrial genes—identified by gene symbols beginning with "MT-"—were filtered out. For pancreatic ductal adenocarcinoma (PDAC), the mitochondrial threshold was adjusted to 6.5%, and for esophageal squamous cell carcinoma (ESCC), the lower UMI threshold was raised to 500 to improve cluster resolution.

These filtering criteria were applied independently to each sample prior to dataset integration. Filtered Seurat objects were then merged by cancer type to generate integrated datasets for downstream analyses.

Batch Correction and Doublet Removal

Following the initial quality control and merging of samples by cancer type, doublets were identified and removed using DoubletFinder (version 2.0.4). This step was applied to five cancer types (PDAC, HCC, BC, GC, and TC); colorectal cancer (CRC) and esophageal squamous cell carcinoma (ESCC) were excluded from doublet filtering due to their pre-integrated or population-sorted structure, respectively. The expected doublet rate was set at 7.5% for all datasets except for breast cancer, where it was increased to 10% to improve cluster separation. The optimal pK value was determined for each dataset using parameter sweep analysis (paramSweep, summarizeSweep, and find.pK functions), while the pN parameter was fixed at 0.25.

To correct for batch effects resulting from multi-sample integration, Harmony (version 1.2.3) was applied to the merged Seurat objects for each cancer type. Harmony integration was performed after doublet removal to minimize technical variation across samples while preserving biologically relevant structure.

Dimensionality Reduction and Clustering

After batch correction and doublet removal, principal component analysis (PCA) was performed to reduce data dimensionality. The top 10 principal components were selected for downstream analysis. Cell-to-cell similarity graphs were constructed using Seurat's FindNeighbors() function based on these components, and clustering was performed using the FindClusters() function with a resolution parameter of 0.5.

Uniform manifold approximation and projection (UMAP) was used for two-dimensional visualization of the data using the first 10 principal components. The resulting clusters were evaluated for consistency with known marker gene expression and overall cell-type identity.

Cell Type Annotation

Cell type annotation was performed manually based on canonical marker gene expression. For well-characterized populations, established markers were used to identify major components of the tumor microenvironment. Cancer cells were defined by expression of EPCAM and KRT18, and T cell subsets were identified by CD3E, CD8A, and FOXP3. Endothelial cells were marked by PECAM1 and RAMP2, and pericytes were characterized by RGS5. Fibroblasts, including cancer-associated fibroblasts (CAFs), were identified by expression of DCN, C1S, CXCL12, and COL12A1. B cells were annotated using MS4A1, mast cells by KIT, myeloid cells by CD14, and plasma cells by MZB1.

For clusters lacking clear marker expression, differentially expressed genes were identified using *FindAllMarkers()* in Seurat, and cluster identity was inferred based on top-ranked genes.

Cell–Cell Communication Analysis

Cell–cell communication analysis was performed using the CellChat package (version 1.6.1) on each cancer type individually. For each dataset, normalized expression data and unsupervised cluster annotations (seurat_clusters) were used to construct CellChat objects. Known ligand–receptor interactions from the CellChatDB.human database were applied, with a focus on the “Secreted Signaling” category.

Overexpressed genes and interactions were identified using the identifyOverExpressedGenes() and identifyOverExpressedInteractions() functions. Communication probabilities between clusters were computed using computeCommunProb() and computeCommunProbPathway(), and clusters with fewer than 10 cells were excluded from analysis. Network-level communication summaries were aggregated using aggregateNet() and visualized using circular plots (netVisual_circle()).

All signaling pathways were examined and visualized individually to assess inter-cluster interactions. For each pathway, ligand–receptor pair contributions were quantified using netAnalysis_contribution(), and communication centrality metrics were computed using netAnalysis_computeCentrality(). The functional roles of clusters as signal senders or receivers were further evaluated using outgoing and incoming signaling role heatmaps.

Analysis of Public Stromal and Prognostic Datasets

Gene expression profiles from microdissected stromal tissues of 13 colorectal cancer tumors were obtained from the publicly available dataset GSE35602, generated using the Agilent 4 × 44K microarray platform. Probe intensities were normalized using a single GAPDH probe (A_23_P13899), and the stromal-to-epithelial expression ratio was calculated. Gene–gene correlation analysis was performed using Pearson correlation coefficients.

Prognostic relevance of CAF-associated genes was evaluated based on clinical and gene expression data from 232 colon cancer samples in the GSE17538 dataset (Affymetrix Human Genome U133 Plus 2.0 Array). These data were used to reference the prognostic impact of individual genes.

Gene Expression and Survival Analysis

We retrieved microarray gene expression profiles from the publicly available dataset GSE15459, which comprises Affymetrix Human Genome U133 Plus 2.0 Array data from 201 primary gastric cancer samples. Raw CEL files (n = 201) were downloaded from the NCBI Gene Expression Omnibus (GEO), of which 200 were successfully processed. Expression data were normalized using the robust multi-array average (RMA) method implemented in the affy package in R (version X.X.X). The final expression matrix included 54,675 probe sets across 200 samples. Clinical outcome data were available for 192 patients with gastric adenocarcinoma, as previously reported by Liu et al. (Gastroenterology, 2013), and were matched with gene expression profiles for further survival analysis.

To adjust for potential technical variability in gene expression, GAPDH was used as a

normalization reference. Among three candidate GAPDH-associated probe sets (213453_x_at, 217398_x_at, and 212581_x_at), we selected 213453_x_at based on having the highest average expression and strong Pearson correlation ($r > 0.98$) with the other two probes. Normalized expression values for target genes were calculated by dividing the linear-scale expression of each probe by that of the selected GAPDH probe, and multiplying by 100 to yield a relative expression score.

We analyzed the expression of CD14, FCN1, and TREM2 using individual probe sets without collapsing multiple probes per gene. Specifically, we used the following probe sets: 201743_at (CD14), 1560034_a_at and 205237_at (FCN1), and 219725_at (TREM2).

Normalized expression values were exported to JMP Pro (SAS Institute) for downstream analysis. For each gene, receiver operating characteristic (ROC) analysis was performed to identify the optimal cutoff value for dichotomizing patients into high- and low-expression groups based on sensitivity and specificity for overall survival status. Kaplan–Meier survival analysis was conducted using these binary groupings. Survival time was defined in months, and death was treated as the event of interest. Patients alive at last follow-up were censored. In the survival status column, death was coded as 1 and censoring (alive) as 0; accordingly, the censoring code in JMP was set to 0.
